# Anemia and frailty in the aging population: implications of dietary fiber intake (findings of the US NHANES from 2007–2018)

**DOI:** 10.1186/s12877-023-04352-9

**Published:** 2023-10-07

**Authors:** HuanRui Zhang, Xuejiao Wei, Jiani Pan, XiTao Chen, XiaoDi Sun

**Affiliations:** 1 Department of Geriatric, The First Hospital of China Medical University, NO.155 Nanjing North Street, Heping Ward, Shenyang, 110001 China; 2 Department of Health Services and Policy Research, Queen’s University, Kingston, ON Canada; 3 Department of Thoracic Surgery, The First Hospital of China Medical University, NO.155 Nanjing North Street, Heping Ward, Shenyang, 110001 China

**Keywords:** Dietary fiber, Anemia, Frailty, Older individuals, NHANES

## Abstract

**Background:**

Frailty has long been seen as an indicator of reduced physical functions in the elderly, which may be caused by a variety of chronic illnesses or cancerous tumors. Dietary fiber was connected with anemia and frailty, whereas it was uncertain if dietary fiber consumption modifies the impact of anemia on frailty in elderly adults.

**Methods:**

We performed a secondary analysis using older adults aged 60 years and over from the National Health and Nutrition Examination Survey (NHANES) 2007–2018 cycles. Dietary fiber intake was estimated using two 24-h dietary recalls. Participants were dichotomized as frail or non-frail based on a modified Fried physical frailty phenotype from previous NHANES studies. The weighted logistic regression was used to estimate the odds ratio (OR) and confidence interval (CI) for the associations between hemoglobin levels and frailty at high- and low-dietary fiber intake levels.

**Results:**

A total of 9644 older adults were included in this study, and the weighted sample was 56,403,031, of whom 3,569,186 (6.3%) were deemed to be frail, and the remainder were deemed to be non-frail. Among the low dietary fiber intake group, higher hemoglobin was significantly associated with a lower risk of frailty (OR = 0.79, 95% CI: 0.71–0.87), and anemia was associated with an almost threefold elevated risk of frailty (OR = 3.24, 95% CI:1.98–5.29) in the fully adjusted model. However, this phenomenon was not observed in groups with high dietary fiber intake. In addition, L-shaped dose response relationship was found in the high dietary fiber intake group (*P* overall association < 0.001; *P* non-linear association = 0.076). Whereas the dose response relationship was not significant in the high dietary fiber intake group (*P* overall association 0.752; *P* non-linear association = 0.734).

**Conclusions:**

Frailty was positively associated with the severity of anemia in older adults with low, but not high, dietary fiber intake. Adequate fiber intake may be an innovative dietary strategy to reduce frailty in older adults.

## Introduction

The outcome of longer life expectancies is an aging world population [1]. Additionally, compared to 1990 (9.2%), there will be 21.3% more people worldwide who are 60 or older by 2050 [2]. The majority of an aging person's organs will start to fail, which may lead to frailty or pre-frailty, then incapacity, and death [3]. Similar to frailty, aging may lead to poor physical and mental health, poor cognitive function, sarcopenia, cardiovascular disease, and an increased risk of falling. Frailty may also exacerbate these risks. However, several chronic illnesses may worsen elderly people's frailty issues and vice versa [4]. On the other hand, frailty may increase a senior's susceptibility to several chronic illnesses. According to a number of studies [5, 6], frailty can also affect the prognosis or course of treatment for particular abnormal disorders. Anemia, which is more common in the elderly than in other age groups and is thought to be brought on by blocked iron trafficking, interleukins 1, 6, and 10, or direct iron deficiency, has been linked to chronic kidney disease (CKD), infections, and cancer [7]. Finding additional potential targets that might have an impact on frailty in older people with anemia is crucial since anemia is widespread in older people and is expected to prevent frailty from getting worse.

Despite the fact that older Japanese adults who follow high-fiber diets are less likely to become frail [8], the lack of a minimum standard for achieving high dietary fiber places a significant burden on research into the relationship between fiber intake and a lower prevalence of frailty as well as efforts to further promote dietary fiber intake. Because there are no strict guidelines on the recommended daily fiber intake, people may consume excessive amounts of fiber to achieve frailty protection, which makes increasing fiber intake difficult in low-income households and places where higher-fiber foods are difficult to get. The transition to high dietary fiber intake for older people, which has been documented, also adds to the difficulties of consuming the right amount of dietary fiber [9, 10]. According to a recent systematic review [11], long-fiber interventions should be used to further examine the role that fiber consumption may play in promoting dietary iron bioavailability and absorption. In a rat model [12], dietary fiber from partially hydrolyzed guar gum increased intestinal iron absorption and hemoglobin regeneration, which may be a useful treatment for iron deficiency anemia. In this cohort examination, which is associated with frailty status in elderly people, the prevalence of frailty increased when dietary fiber consumption decreased, according to a multicenter study [13]. However, it is still unknown if dietary fiber intake affects the relationship between anemia and frailty in elderly people. Based on the National Health and Nutrition Examination Survey (NHANES) database in the US, the goal of our inquiry is to ascertain whether there would be a substantial link between anemia and frailty impacted by dietary fiber consumption among the aging population.

## Methods

### Study design and patients

We performed a secondary analysis of the National Health and Nutrition Examination Survey (NHANES) 2007–2018 cycles. NHANES is a complex, multistage, and probabilistic sampling design survey conducted annually and released biannually by the National Center for Health Statistics (NCHS). More details about NHANES survey procedures are available at https://www.cdc.gov/nchs/index.htm. The NCHS Ethics Review Board approved the protocol, and all participants provided an informed consent form (https://www.cdc.gov/nchs/nhanes/irba98.htm).

**Fig. 1:**
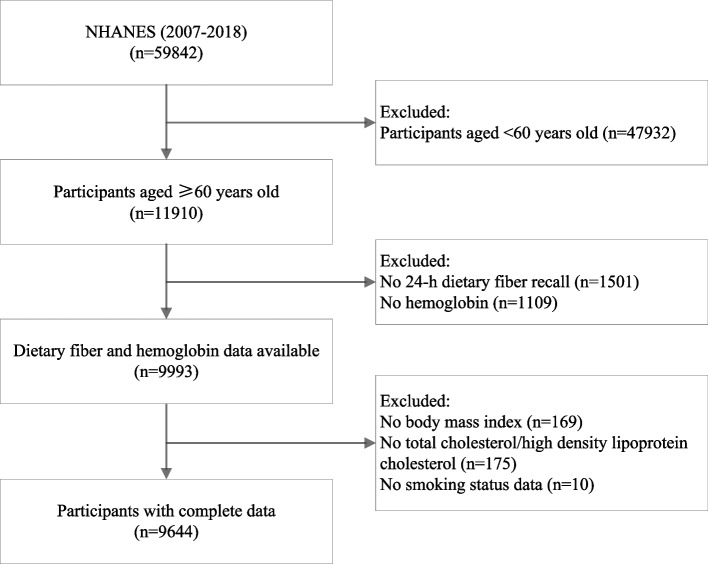
Flow chart of sample selection

The present study data were derived from NHANES 2007–2018 (*N* = 59842). Participants aged 60 years and over with at least one reliable 24-h dietary recall, hemoglobin and frail related data were included (*N* = 9993). After further excluding those without complete data for the model covariates, 9644 participants were considered eligible for final analysis (Fig. 1).

### Dietary fiber intake

Two 24-h dietary recalls were used to assess dietary intake during the 24-h period prior to the interview. The first 24-h dietary survey was conducted by trained researchers in the Mobile Examination Center, and the second was collected by telephone 3–10 days later. The US Department of Agriculture’s Food and Nutrient Database for Dietary Studies was used to calculate dietary fiber intake from all foods and beverages. Dietary fiber intake was calculated by averaging two-day dietary recall data if participants had two-day data available. Otherwise, a single dietary recall was used.

### Modified frailty assessment

Frailty was defined using a modified Fried physical frailty phenotype from previous NHANES studies [14, 15], which is an acceptable alternative definition in large research studies when no direct physical measurement can be used [16, 17]. Criteria included weakness, low physical activity, exhaustion, slow walking speed, and unintentional weight loss. Weakness was defined as self-reported lifting or carrying difficulty. Low physical activity was defined as the highest quintile of self-reported minutes of sedentary time by age groups. Exhaustion was defined as self-reported tiredness or a lack of energy. Slow walking speed was defined as self-reported walking difficulty between rooms on the same floor. Unintentional weight loss was defined as self-reported unintentional weight loss (≥ 10 lbs) in the past one year. Frail was defined as having 3–5 criteria.

### Covariates

Age, gender, ethnicity (Non-Hispanic White, Non-Hispanic Black, Hispanic, and other races), education (less than high school and above high school), marital status (married/with partner and widowed/divorced/separated/single), smoking (never smoking, former smoking, and current smoking), body mass index (BMI), serum lipid level, hypertension, diabetes, cardiovascular disease (CVD), and self-reported history of using lipid-lowering drugs, antihypertensive drugs, hypoglycemic agents, and antiplatelet drugs) were considered as confounders. The serum lipid level was defined as the ratio of total cholesterol to high-density lipoprotein cholesterol. Hypertension was defined as blood pressure ≥ 140/90 mmHg, self-reported, or taking antihypertensive medications. Diabetes was defined as a fasting glucose ≥ 126 mg/dL, self-reported, or taking any oral hypoglycemic agent or insulin. CVD was defined as the self-reported diagnosis of heart failure, coronary heart disease, angina, heart attack or stroke.

### Statistical analysis

Considering the complex multistage sampling design of NHANES, an appropriate sampling weight (1/6*WTDRD1) was constructed based on NHANES guidance. Descriptive statistics were presented for demographic and clinical characteristics by frailty groups. The continuous variables were expressed as a weighted mean (standard error, SE) and tested by *t*-test. The categorical variables were described as weighted proportions and compared via the Rao-Scott chi-square test. The weighted logistic regression was used to estimate the odds ratio (OR) and confidence interval (CI) for the associations between dietary fiber intake and hemoglobin and frailty. Furthermore, to test whether the association differed between high and low dietary fiber intake levels, the study sample was divided into high- and low-dietary fiber intake subgroups, and associations were estimated in subgroups. In Model 1, age, gender, and ethnicity were adjusted. Model 2 incorporated additional adjustments for marital status, education, BMI, and smoking status. Furthermore, in Model 3, confounding factors such as total cholesterol/high density lipoprotein cholesterol, hypertension, diabetes, cardiovascular disease, antihypertensive drugs, hypoglycemic agents, lipid-lowering drugs, and antiplatelet drugs were considered for further adjustments. In addition, restricted cubic splines were also depicted to assess the dose–response relationship between hemoglobin and frailty in diverse subgroups. Finally, a weighted logistic regression was performed to identify risk factors associated with dietary fiber intake levels. Analyses were performed using R version 4.0.3, and *P* < 0.05 was considered statistical significance.

## Results

### The demographic and clinical characteristics of participants

The present analysis comprised 9644 older adults with complete data from NHANES 2007–2018. The weighted demographic and clinical characteristics of participants based on an 8-year weight (1/6*WTDRD1) are presented in Table 1. The weighted sample was 56,403,031, of whom 3,569,186 (6.3%) were deemed to be frail, and the remainder were deemed to be non-frail. The weighted average age was 69.65, and 54.3% of them were female. In the frail group, age, BMI, the proportions of married/with partner, below high school education, current smoking, hypertension, diabetes, cardiovascular disease, antihypertensive treatment, hypoglycemic treatment, lipid-lowering treatment, and antiplatelet treatment were significantly higher than the non-frail group (All *P* values < 0.05). In addition, hemoglobin and dietary fiber intake levels were significantly lower in the frail group (All *P* values < 0.05).

**Table 1:** Baseline characteristics of weighted sample by frail and non-frail groups

Characteristics	Total(*n* = 9644)	Non-frail(*n* = 8945)	Frail(*n* = 699)	*P*-value
Weighted number	56,403,031	52,833,845	3,569,186	
Age, years	69.65 (0.12)	69.48 (0.12)	72.18 (0.40)	< 0.001
Gender, female	54.3 (0.54)	53.8 (0.50)	62 (3.07)	0.009
Ethnicity				0.149
Non-Hispanic White	78.4 (1.22)	78.5 (1.23)	76.1 (2.33)	
Non-Hispanic Black	8.1 (0.65)	8 (0.66)	10.4 (1.23)	
Mexican American/Hispanic	3.6 (0.40)	3.6 (0.39)	3.9 (0.76)	
Other	9.9 (0.70)	9.9 (0.70)	9.5 (1.44)	
Education, above high school	82.2 (0.82)	83 (0.81)	70.3 (0.22)	< 0.001
Marital status, married/with partner	64.4 (0.86)	65.4 (0.84)	48.6 (2.63)	< 0.001
Smoking				< 0.001
Never	50.1 (0.73)	50.6 (0.77)	41.9 (2.07)	
Former	39.1 (0.63)	39 (0.67)	40.8 (2.47)	
Current	10.8 (0.46)	10.4 (0.50)	17.4 (2.35)	
Body mass index, kg/m2	29.24 (0.11)	29.10 (0.11)	31.44 (0.40)	< 0.001
Total cholesterol /High density lipoprotein cholesterol	3.74 (0.02)	3.74 (0.02)	3.73 (0.07)	0.844
Hypertension diagnosis	67.6 (0.83)	66.6 (0.88)	80.9 (2.39)	< 0.001
Diabetes diagnosis	25.8 (0.64)	24.8 (0.69)	40 (2.66)	< 0.001
Cardiovascular disease diagnosis	22.2 (0.63)	20.9 (0.64)	41.3 (2.53)	< 0.001
Antihypertensive treatment	51.6 (0.90)	50.4 (0.97)	68.5 (2.26)	< 0.001
Hypoglycemic treatment	17.4 (0.57)	16.5 (0.62)	31.5 (2.55)	< 0.001
Lipid-lowering treatment	41.8 (0.76)	41.5 (0.75)	47.3 (2.80)	0.035
Antiplatelet treatment	7 (0.40)	6.6 (0.41)	11.8 (1.55)	< 0.001
Hemoglobin, g/dL	14.00 (0.03)	14.04 (0.03)	13.43 (0.09)	< 0.001
Dietary fiber intake, g/day	16.98 (0.17)	17.15 (0.18)	14.43 (0.41)	< 0.001
All continuous variables were presented as weighted mean(SE) and categorical variables were presented as weighted %(SE)				

### Association of hemoglobin and dietary fiber intake with frail

Model 1 showed that hemoglobin was associated with a lower risk of frailty (OR = 0.79; *P* < 0.001), and an increase in dietary fiber intake was correlated with a lower risk of frailty (OR = 0.96; *P* < 0.001) in Table 2. The associations of hemoglobin and dietary fiber intake with frailty were still significant in further adjusted model 2 (*P* < 0.001; *P* = 0.005) and model 3 (*P* < 0.001; *P* = 0.010).

**Table 2:** Weighted association of the hemoglobin and dietary fiber intake with frail

Variables	Model 1		Model 2		Model 3	
	OR(95% CI)	*P* value	OR(95% CI)	*P* value	OR(95% CI)	*P* value
Hemoglobin (g/dL)	0.79(0.73, 0.87)	< 0.001	0.77(0.71, 0.84)	< 0.001	0.81(0.74, 0.89)	< 0.001
Dietary fiber intake (g/day)	0.96(0.94, 0.98)	< 0.001	0.97(0.96, 0.99)	0.005	0.97(0.96, 0.99)	0.01

Model 1 adjusted for age, gender and ethnicity

Model 2 adjusted for Model 1 plus marital status, education, BMI, smoking status

Model 3 adjusted for Model 2 plus total cholesterol/high density lipoprotein cholesterol, hypertension, diabetes, cardiovascular disease, antihypertensive drugs, hypoglycemic agents, lipid-lowering drugs, antiplatelet drugs

OR (95% CI), odds ratio and 95% confidence interval

### Association between hemoglobin and frailty in the high and low dietary fiber intake groups

Among the low dietary fiber intake group, with adjustments applied for age, gender, and ethnicity, marital status, education, BMI, smoking status, total cholesterol/high-density lipoprotein cholesterol, hypertension, diabetes, cardiovascular disease, antihypertensive drugs, hypoglycemic agents, lipid-lowering drugs, and antiplatelet drugs, higher hemoglobin was significantly associated with a lower risk of frailty (Continuous: OR = 0.79, *P* < 0.001; Q2 vs. Q1: OR = 0.60, *P* = 0.002; Q3 vs. Q1: OR = 0.53, *P* < 0.001; Q4 vs. Q1: OR = 0.51, *P* = 0.006;), and anemia was associated with an almost threefold elevated risk of frailty (OR = 3.24, *P* < 0.001) (Table 3). However, the associations above disappeared in the high dietary fiber intake group in all multivariate adjusted models (All *P* values > 0.05) (Table 3).

**Table 3:** Weighted association between hemoglobin and frail in the high and low dietary fiber intake groups

Variables	Weighted sample *n* = 56,403,031	Model 1		Model 2		Model 3	
		OR(95% CI)	*P* value	OR(95% CI)	*P* value	OR(95% CI)	*P* value
High dietary fiber intake (*n* = 2287)							
Hemoglobin, g/dL							
Continuous	14,040,842	0.92(0.76, 1.11)	0.374	0.89(0.72, 1.09)	0.251	0.97(0.79, 1.20)	0.811
Quartile							
Q1 (< 13.1)	2,383,943	Ref		Ref		Ref	
Q2 (13.1–14.0)	3,598,798	1.04(0.51, 2.13)	0.916	0.99(0.46, 2.13)	0.989	1.06(0.50, 2.28)	0.873
Q3 (14.0–14.9)	3,683,031	1.02(0.56, 1.87)	0.946	0.97(0.50, 1.88)	0.938	1.25(0.61, 2.53)	0.546
Q4 (≥ 14.9)	4,375,070	0.65(0.28, 1.50)	0.313	0.57(0.23, 1.41)	0.227	0.77(0.31, 1.93)	0.577
P for trend			0.346		0.259		0.68
Anemia							
No	13,904,846	Ref		Ref		Ref	
Yes	135,996	1.04(0.32, 3.36)	0.943	1.21(0.37, 4.01)	0.752	0.96(0.26, 3.56)	0.947
Low dietary fiber intake (*n* = 7357)							
Hemoglobin, g/dL							
Continuous	42,362,189	0.77(0.70, 0.86)	< 0.001	0.76(0.69, 0.84)	< 0.001	0.79(0.71, 0.87)	< 0.001
Quartile							
Q1 (< 13.1)	10,372,644	Ref		Ref		Ref	
Q2 (13.1–14.0)	10,798,568	0.54(0.40, 0.74)	< 0.001	0.53(0.39, 0.74)	< 0.001	0.60(0.44, 0.81)	0.002
Q3 (14.0–14.9)	10,221,403	0.51(0.37, 0.72)	< 0.001	0.48(0.34, 0.68)	< 0.001	0.53(0.37, 0.74)	< 0.001
Q4 (≥ 14.9)	10,969,574	0.49(0.31, 0.80)	0.005	0.44(0.28, 0.69)	0.001	0.51(0.32, 0.81)	0.006
P for trend			0.003		< 0.001		0.002
Anemia							
No	41,540,453	Ref		Ref		Ref	
Yes	821,736	3.36(2.04, 5.56)	< 0.001	3.63(2.15, 6.14)	< 0.001	3.24(1.98, 5.29)	< 0.001

Model 1 adjusted for age, gender and ethnicity

Model 2 adjusted for Model 1 plus marital status, education, BMI, smoking status

Model 3 adjusted for Model 2 plus total cholesterol/high density lipoprotein cholesterol, hypertension, diabetes, cardiovascular

disease, antihypertensive drugs, hypoglycemic agents, lipid-lowering drugs, antiplatelet drugs

High dietary fiber intake was defined fiber intake ≥ 21.30 g/day and low dietary fiber intake was defined fiber intake < 21.30 g/day

OR (95% CI), odds ratio and 95% confidence interval

### Dose–response relationships differed by dietary fiber intake levels

**Fig. 2:**
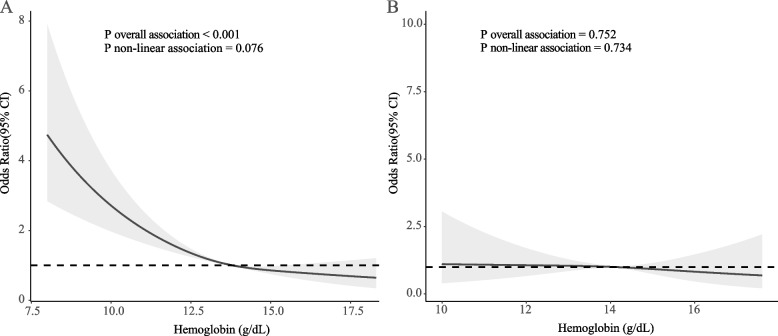
Associations of hemoglobin levels and frail using restricted cubic spline models in low- (**A**) and high- (**B**) dietary fiber intake groups. All models were adjusted for age, gender and ethnicity, marital status, education, BMI, smoking status, total cholesterol/high density lipoprotein cholesterol, hypertension, diabetes, cardiovascular disease, antihypertensive drugs, hypoglycemic agents, lipid-lowering drugs, antiplatelet drugs

As depicted in Fig. 2A, an L-shaped dose–response relationship was found between hemoglobin and the risk of frailty in the low dietary fiber intake group (*P* overall association < 0.001; *P* non-linear association = 0.076), and the relationship above began to flatten when hemoglobin was higher than 13.8 g/dL. Whereas the dose response relationship was not significant in high dietary fiber intake group (*P* overall association = 0.752; *P* non-linear association = 0.734) (Fig. 2B).

### Hemoglobin differences between dietary fiber intake levels

As shown in Table 4, compared to high dietary fiber intake, hemoglobin (13.94 vs. 14.21; *P* < 0.001) was at a lower level in the low dietary fiber intake group.

**Table 4:** Weighted difference of hemoglobin in the high and low dietary fiber intake groups

Variable	Low dietary fiber intake	High dietary fiber intake	*P* value
Hemoglobin, g/dL	13.94 (0.03)	14.21 (0.05)	< 0.001

High dietary fiber intake was defined fiber intake ≥ 21.30 g/day and low dietary fiber intake was defined fiber intake < 21.30 g/day. Hemoglobin was presented as weighted mean(SE)

### Association between demographics and dietary fiber intake levels

**Fig. 3:**
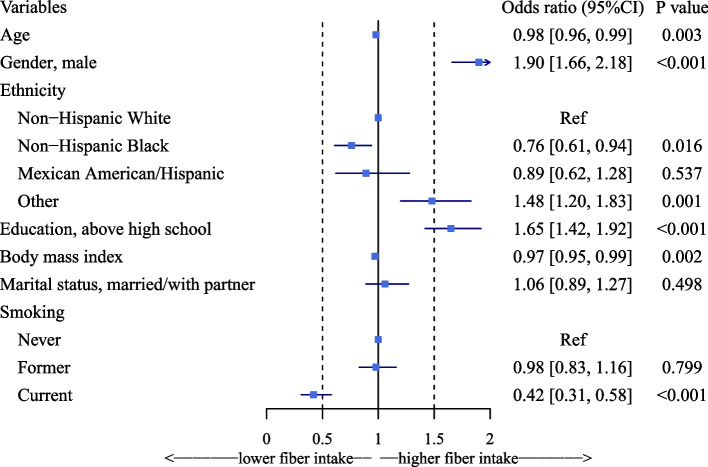
Forest plot of associations between demographics and dietary fiber intake levels

Multivariable logistic regression results are presented in Fig. 3. In multivariable logistic regression analysis, demographics found to be associated with higher dietary fiber intake levels were male (OR = 2.02, *P* < 0.001), other ethnicity (OR = 1.54, *P* < 0.001), and above high school (OR = 1.65, *P* < 0.001). Other demographics associated with lower odds of having a high dietary fiber intake level were older (OR = 0.98, *P* = 0.002), Non-Hispanic Black (OR = 0.69, *P* = 0.001), higher BMI (OR = 0.97, *P* < 0.001), and current smoking (OR = 0.37, *P* < 0.001).

## Discussion

The study, based on a nationally representative sample of older individuals in the United States from the National Health and Nutrition Examination Survey (NHANES), examined whether sufficient dietary fiber consumption moderated the correlation between anemia and frailty. In comparison to older individuals without anemia, those with severe anemia were substantially more frailty. This study revealed that older individuals with frailty had lower hemoglobin levels and fiber consumption than older adults without frailty. Notably, the correlation between anemia and frailty in elderly adults varies by dietary fiber consumption, compared to older adults without anemia. In older individuals with low dietary fiber consumption, anemia was associated with a higher frailty risk compared to those without anemia, but no association was detected in those with high dietary fiber intake. The higher the severity of anemia, the higher the risk of frailty. However, this trend was not observed in individuals with high fiber consumption. The study revealed that a high intake of dietary fiber may have neglected the negative effect of anemia on elderly frailty.

Several prospective cohort studies [18, 19] have established the causal relationship between anemia and frailty. The Swedish National Study [19] on Aging and Care in Kungsholmen demonstrated a substantial correlation between frailty and cardiovascular disease, anemia, and dementia during a 12-year period. 11.1% of the 2,122 individuals in the 6-year study developed frailty, which was associated with a 2.25-fold greater risk for cardiovascular disease, anemia, and dementia. The prospective trials conducted in China [20] and Spain [18, 21] revealed comparable outcomes. There is evidence that anemia influences a range of chronic diseases [22, 23] including cancer [24], which are the phenotypes of aged frailty. With a reported prevalence of 10% to 20% in older adults, anemia is a frequent clinical disorder that may have both hemodynamic and nonhemodynamic effects on cardiovascular disease (CVD). Anemia is a marker for increased risk of major adverse cardiovascular events (MACE) in the population besides those with heart failure [23]. A cohort analysis [24] found that the incidence of cancer in the anemic group was 3% higher than in the control group (hazard ratio = 1.03; 95% confidence range = 1.01 to 1.05; *P* = 0.023). Independently, anemia was related to a higher overall risk of cancer. In accordance with a previous study, our results demonstrated that anemia was substantially related to increased frailty in the elderly with low dietary fiber intake compared to those with high dietary fiber intake. There is no additional reduction in the risk of frailty once anemia has been eliminated, even if hemoglobin levels continue to rise. The correlation between frailty and anemia might be explained by the relationship between hemoglobin levels and frailty risk.

While we may not neglect the fact that dietary fiber intake varied by several factors throughout our study, including gender, ethnicity, marriage status, current smoking, and educational level (Fig. 3), Increased dietary fiber consumption was associated with higher dietary fiber intake in males, those with a high school diploma or higher, and married individuals. In contrast, low fiber intake was positively associated with the black race and current smoking. These features of the natural distribution of dietary fiber intake will help us more accurately target the intended population, offer preventative measures for dietary fiber intake, and reduce the risk of frailty mostly through dietary fiber intake intervention.

Previous studies confirmed that high dietary fiber intake reduced the risk of anemia [9, 25, 26]. Compared to the Low Fiber grouping, the High Fiber group had increased iron levels (35 vs. 21 mg/day) and a decreased risk of anemia (10% vs. 25%) in the prospective cross-sectional research [9]. In a prior work [25], the effects of feeding soy fiber on gastrectomy-induced iron malabsorption, anemia, and exercise performance impairment in rats were investigated. In rats given a WSSF (water-soluble soybean fiber) diet (50 g/kg diet), iron absorption and hematological factors of the gastrectomized rats were equivalent to those of the sham-operated rats, suggesting that WSSF consumption improves iron absorption and avoids anemia following gastrectomy. In a combined study [26], the GDQS (Global Diet Quality Score) was substantially and marginally rank-correlated with fiber intakes (*r* = 0.22 for men and 0.25 for women), which was strongly linked to better diet, more hemoglobin, and a lower risk of anemia.

There is a negative correlation between dietary fiber consumption and several debilitating conditions in senior citizens. A recent randomized single-blind study [27] showed that supplementing with dietary fiber for 4 weeks can lower residents of long-term care facilities’ use of laxatives and may be a useful way to treat constipation. Residents’ frailty can be lessened by either lowering drug use or treating constipation. A meta-analysis [28] revealed that people with IBD (inflammatory bowel disease) consume considerably less dietary fiber than healthy individuals. Meanwhile, individuals with IBD are more susceptible to malnutrition, micronutrient deficiencies, anemia, and osteoporosis if they fail to consume a healthy diet. Accordingly, a high dietary fiber intake may lower the risk of overall mortality. Seven prospective cohort studies [29] found that people who consumed the most fiber had a hazard ratio of 0.77 (95% confidence interval: 0.74–0.81) in frailty compared to those who consumed the least fiber. From 2009 to 2014, the National Health and Nutrition Examination Survey (NHANES) [30] discovered that the quantity of total fiber and cereal fiber in a person's diet was associated with a decreased risk of hyperuricemia, applying thresholds of 7.0 mg/dL for men and 6.0 mg/dL for women. A dose–response meta-analysis [31] of the link between dietary fiber intake and ovarian cancer risk revealed a substantial negative correlation (an increase of 10 g/day; combined RR: 0.88; 95% CI: 0.82, 0.93) between dietary fiber intake and ovarian cancer risk. Zheng et al. [32] discovered that consuming more whole grains overall was linked to a lower incidence of bladder cancer (BC). High consumption of whole grains and dietary fiber is related to a lower incidence of insulin sensitivity, hyperglycemia, and inflammatory processes, which are recognized cancer risk factors. Numerous studies [33–35] have demonstrated that dietary fiber consumption is linked to malignancies and chronic illnesses, which raise the risk of frailty in the elderly. Increased dietary fiber consumption [36] lowers the risk of acquiring a number of chronic illnesses, including type 2 diabetes, cardiovascular disease, and various malignancies, and has been linked to a lower body weight. The average American consumes 17 g of dietary fiber per day, with just 5% of the population achieving the recommended amount. Dietary fiber consists of a complex collection of non-digestible compounds, mostly polysaccharides. Multiple epidemiological studies [37] have shown statistically significant decreases in the risks of obesity, type 2 diabetes, cardiovascular disease, colon cancer, and premenopausal breast cancer with greater fiber intakes. A high intake of fiber may increase gut hormones, which may operate through pathways including G-protein-coupled receptors (GPRs), histone deacetylase (HDAC), and aromatase enzymes. Consequently, fiber consumption results in reduced glucose levels and insulin sensitivity, thereby decreasing the incidence of T2DM, CVD, and some malignancies.

However, no prior research has examined whether dietary fiber may affect the association between anemia and frailty. Based on this prior research, our study expanded on these results by evaluating how dietary fiber alters the connection between anemia and cognitive performance. Our investigation indicated negative connections between older individuals' hemoglobin levels and frailty. The findings revealed that the link between hemoglobin level and frailty was inconsistent at various levels of dietary fiber consumption, suggesting that the detrimental relationship between uncontrolled anemia and frailty may be mitigated by dietary fiber intake. Increasing dietary fiber consumption may be a possible treatment strategy for frailty in elderly individuals with uncontrolled anemia.

The risk of anemia nearly tripled among those who were frail. In order to develop nutritional therapies that successfully reduce frailty, it is necessary to pinpoint the specific problems connected to the dietary practices of individuals based on sex differences, as stated in a Japanese observational study [8]. If they consumed less soluble dietary fiber, potassium, folate, and vitamin C, men and women were more likely to be frail. Only 35.5% of the Japanese individuals consumed enough dietary fiber, and more than 80% of them acquired adequate protein, copper, vitamin B12, and pantothenic acid. In this study, 88.9% of participants had excessive salt intake, and less than 90% had appropriate fiber intake [38, 39]. The British Regional Heart Study [40], including 945 men aged 70 to 92 years old without prevalent frailty, implied that adherence to a Mediterranean-style diet was connected with a decreased risk of frailty in older people. A high-fat, low-fiber diet pattern increased the risk of frailty. Previous studies [41] have demonstrated that the hepcidin hormone, which controls iron homeostasis by proteolytically destroying ferroportin, the only identified iron exporter in mammals, modulates iron bioavailability during pregnancy. Low blood hepcidin levels enhance dietary iron translocation across the placenta and maternal iron absorption, which enhances neonatal iron status. Blood hepcidin concentrations in pregnant women were positively correlated with the quantity of fiber they consumed. According to a study from the NHANES database [42], a substantial proportion of young children are at risk of inadequate fiber intake. Despite adequate iron intake, serum ferritin and hemoglobin levels indicated that 7.4% and 2.5% of patients, respectively, displayed iron deficiency and anemic symptoms. Based on this analysis, it appears that dietary fiber may control anemia in ways other than influencing iron absorption. Lustgarten MS [43] discovered that a high-fiber diet may be a key strategy for enhancing the kidney-gut-muscle axis in ESRD (end-stage renal disease) patients and healthy older people. By fermenting dietary fiber, gut bacteria produce the short-chain fatty acids (SCFAs) acetate, propionate, and butyrate. Since excessive Wnt signaling is typically observed in colorectal cancer, it is conceivable that larger intakes of dietary fiber and the fermentation product butyrate protect against the illness by regulating the Wnt pathway, as demonstrated by a randomized controlled experiment [44]. The underlying mechanisms of dietary fiber in modulating chronic diseases and cancers, which increase the risk of frailty, have been demonstrated in prior research. Dietary fiber may have a more significant influence on enhancing frailty performance in elderly adults with untreated anemia compared to those without anemia. However, the precise mechanism remains unknown and requires additional investigation. There is a health advantage to dietary fiber, but the reality is that the global daily consumption of dietary fiber is woefully insufficient. The average consumption of dietary fiber in the United States, the United Kingdom, and China is much less than the 25–35 g/day recommended by the World Health Organization. In our research, the average consumption of dietary fiber was lower than that of those without anemia; elderly individuals with untreated anemia had a much lower consumption of dietary fiber. The findings showed that adequate fiber consumption should be considered a nutritional intervention to minimize the incidence of frailty in elderly adults with untreated anemia.

Our study has several strengths. Compared to elderly adults without anemia, those with severe anemia were much more frailty than their counterparts. This tendency was not noticed among those with a high fiber intake. Analyzing older individuals with and without anemia, the connection between anemia and frailty varied significantly according to fiber intake. In addition, subsequent research revealed that anemia status and dietary fiber intake interacted to promote frailty. The research found that a high consumption of dietary fiber may have mitigated the harmful impact of anemia on geriatric frailty. The findings suggest that the severity of anemia in relation to hemoglobin levels is associated with an increased risk of frailty in the elderly. However, this link will disappear in the absence of anemia. Therefore, we can conclude that a sufficient intake of dietary fiber may be required to avoid frailty in elderly patients with anemia. This investigation may provide a unique nutrition management technique for reducing frailty in older people with anemia. Health officials have to implement nutrition screening to increase fiber intake.

Several limitations of this study must be acknowledged. Due to the cross-sectional nature of the study, it was not possible to determine a causal relationship between dietary fiber intake, anemia, and frailty in the elderly. Measurement errors caused by 24-h dietary recalls may cause biases in target dietary fiber intake estimates. Furthermore, we cannot exclude the possibility that chronic diseases or cancers have a potential influence on anemia, which may cause frailty. More research is required to confirm our findings and investigate their sources and implications. In addition, many iron deficiency indicators associated with frailty, such as serum ferritin [45], soluble transferrin receptors (sTfR) [46] or transferrin saturation [47], were not studied in the NHANES database in the elderly. These factors should be considered in future studies.

## Conclusion

The results indicate that the severity of anemia in relation to hemoglobin levels is connected with an increased risk of frailty in the elderly. A high intake of dietary fiber can minimize its adverse effects. It was proposed that appropriate dietary fiber intake may be essential to preventing frailty in anemic elderly adults. This study could offer a novel nutrition management strategy for preventing frailty in elderly individuals with anemia. Health administrators should employ nutrition screening to boost fiber consumption. Future prospective and interventional investigations are required to validate our findings.

## Data Availability

All data were included in NHANES database. For more information, please visit the official website of NHANES: https://www.cdc.gov/nchs/nhanes/index.htm.
